# Arbuscular Mycorrhizal Symbiosis Improves Ex Vitro Acclimatization of Sugarcane Plantlets (*Saccharum* spp.) under Drought Stress Conditions

**DOI:** 10.3390/plants12030687

**Published:** 2023-02-03

**Authors:** José Luis Spinoso-Castillo, María del Rosario Moreno-Hernández, Eucario Mancilla-Álvarez, Lino Sánchez-Segura, Ricardo Sánchez-Páez, Jericó Jabín Bello-Bello

**Affiliations:** 1 Postgraduate College-Campus Cordoba, Km 348 Cordoba-Veracruz Federal Highway, Amatlan de los Reyes, Veracruz 94953, Mexico; 2 CINVESTAV, Department of Biotechnology and Biochemistry, Irapuato Unit, Km 9.6 North Beltway Highway Irapuato-Leon, Irapuato, Guanajuato 36821, Mexico; 3 CONACYT-Postgraduate College-Campus Cordoba, Km 348 Cordoba-Veracruz Federal Highway, Amatlan de los Reyes, Veracruz 94953, Mexico

**Keywords:** *Glomus intraradices*, chlorophyll, carotenoids, osmolytes, antioxidant capacity

## Abstract

The symbiotic associations between arbuscular mycorrhizal fungi (AMF) and plants can induce drought stress tolerance. In this study, we evaluated the effect of *Glomus intraradices*, a mycorrhizal fungus, on the ex vitro development and survival of sugarcane plantlets subjected to drought stress during the acclimatization stage of micropropagation. In vitro obtained sugarcane plantlets (*Saccharum* spp. cv Mex 69–290) were inoculated with different doses of *G. intraradices* (0, 100, and 200 spores per plantlet) during greenhouse acclimatization. Sixty days after inoculation, plantlets were temporarily subjected to drought stress. We evaluated the survival rate, total chlorophyll, total protein, carotenoids, proline, betaine glycine, soluble phenolic content, and antioxidant capacity every 3 days for 12 days. Symbiotic interaction was characterized by microscopy. Our results showed that the survival rate of inoculated plants was higher in 45% than the treatment without mycorrhizae. Total chlorophyll, protein, proline, betaine glycine content, and antioxidant capacity were increased in AMF inoculated plants. The soluble phenolic content was higher in non-inoculated plants than the treatment with mycorrhizae during the drought stress period. Microscopy showed the symbiotic relationship between plant and AMF. The early inoculation of 100 spores of *G. intraradices* per sugarcane plantlet during the acclimatization stage could represent a preconditioning advantage before transplanting into the field and establishing basic seedbeds.

## 1. Introduction

Sugarcane (*Saccharum* spp. Hybrids), of the Poaceae family, is cultivated in tropical and subtropical areas around the world [1,2]. The economic importance of this species derives from its multiple products, co-products, and by-products, which are used in the food, energy, chemical, and pharmaceutical industries [3,4]. However, sugarcane faces various productivity and competitiveness challenges. Additionally, sugarcane yield is directly affected by the drought caused by climate change [5]. The physiologic effects of drought stress include decreased photosynthesis, loss of turgor, reduced nutrient absorption, and production of reactive oxygen species (ROS). These effects result in oxidative damage, leading to the cell death of plant tissues [6,7]. Plants tolerate drought stress through different physiologic, biochemical, and genetic mechanisms, such as changes in stomatal and photosynthesis regulation, expression of chaperon, channel, and transporter proteins [8,9], osmotic adjustment through the synthesis of compatible osmolytes [10], and increased antioxidant capacity due to higher activity of their antioxidant enzymes (superoxide dismutase, catalases, peroxidases or dismutases) and non-enzymatic antioxidants (e.g., ascorbate, glutathione or carotenoid), which decreases ROS accumulation [11,12].

Biotechnology offers alternatives against low crop productivity, such as micropropagation through plant tissue culture. Micropropagation is important for producing plants with high genetic and phytosanitary quality. Acclimatization, the final stage of micropropagation, consists of the gradual transfer of plantlets from in vitro to ex vitro conditions [13]. This process represents an opportunity to inoculate beneficial microorganisms before transplantation into field conditions [14]. The ecological interactions (commensalism, agonism, amensalism, antagonism, parasitism, and mutualism) between different organisms can be applied in plant and microbial biotechnology, and some of these interactions may be beneficial for all partners involved (mutualistic interactions), whereas others are detrimental for at least one partner (antagonistic interactions) [15,16].

Mycorrhizae have an important role in the development, survival, and success of the species that comprise them (plants and fungi) [17,18]. Arbuscular mycorrhizal fungi (AMF) are obligate biotrophs that form endosymbiotic associations with the roots of vascular plants, including various crops [19,20]. AMF enhance the mineral nutrient uptake [21,22] and photosynthesis of host plants [22], decrease disease invasion [23], induce tolerance to extreme temperature changes [24], reduce heavy metal toxicity [25], and confer drought stress tolerance. The positive effects of AMF on antioxidant [26], polyamine production [27], osmotic adjustment [28], plant development [29], drought tolerance in host plants [30], and water transport [31] have been previously reported in various plants exposed to drought. Additionally, the positive effects of AMF have also been demonstrated in various agri-food crops, such as sugarcane (*Saccharum* spp.) [32], melon (*Cucumis melo*) [33], apple (*Malus prunifolia* (Willd.) Borkh.) [34], common marigold (*Calendula officinalis* L.) [35], and woody plant (*Cinnamomum migao*) [36]. AMF have also been used to mitigate climate change effects, such as drought and salinity. In basil (*Ocimum basilicum*) plants, the effect of drought in open-field was alleviated by inoculation with AMF [37]. In olive (*Olea europaea* L.) plants under greenhouse and water-deficit stress, the plant growth was stimulated by inoculation with *Rhizophagus irregularis*, [38], and in stevia (*Stevia rebaudiana* Bertoni) plants the effect of salt stress induced with NaCl under greenhouse conditions was alleviated with AMF [39]. This study aimed to evaluate the effect of different doses of the mycorrhizal fungus *Glomus intraradices* on the ex vitro development of sugarcane plantlets subjected to drought stress during the acclimatization stage.

## 2. Results

### 2.1. Substrate Matric Potential Measurements

The substrate matric potential (Ψ) increased with increasing days without irrigation, with values of Ψ = –0, –12, –32, –53, and –199 kPa for 0, 3, 6, 9, and 12 days without irrigation, respectively.

### 2.2. Survival Rate and Mycorrhizal Characterization

**Figure 1:**
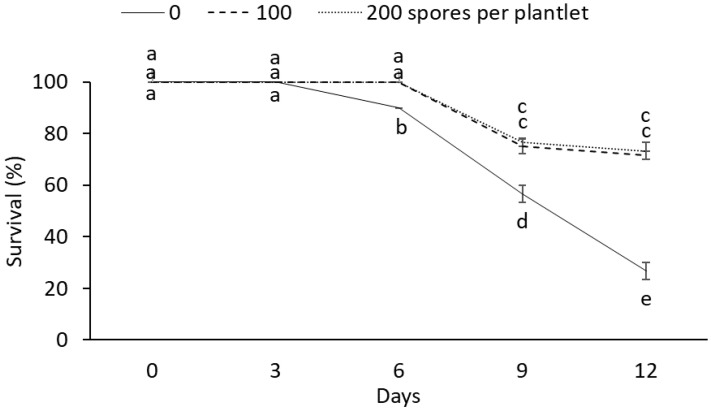
Effect of different doses (0, 100, and 200 spores per plantlet) of arbuscular mycorrhizal fungi (*Glomus intraradices*) on the survival rate of sugarcane plantlets (*Saccharum* spp. cv Mex 69-290) evaluated under drought stress during acclimatization stage. Results are shown as mean ± standard error. Means with different letters (a–e) are significantly different (Tukey, *p* < 0.05).

**Figure 2:**
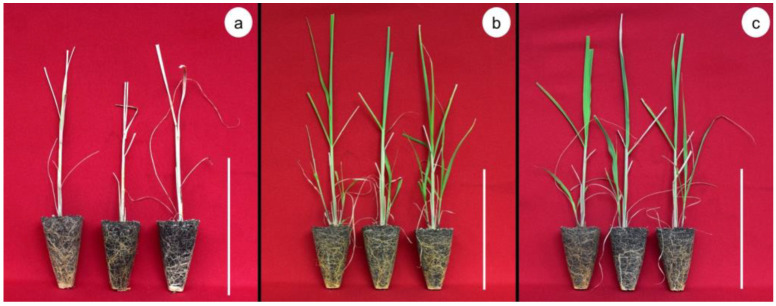
Effect of arbuscular mycorrhizal fungi (*Glomus intraradices*) on survival rate of sugarcane plantlets (*Saccharum* spp. cv Mex 69-290) evaluated under drought stress during acclimatization stage. (**a**) Control treatment, (**b**) 100 spores per plantlet, and (**c**) 200 spores per plantlet. White Bar = 30 cm.

These results show that mycorrhizae affect the survival rate (Figure 1). After six days, drought stress had a negative effect on the survival rate of plantlets without mycorrhizae during the drought stress period, while AMF-inoculated plantlets showed a 100% survival. The highest survival rates were observed in the AMF-treated plantlets after 9 and 12 d under drought stress conditions. Meanwhile, the lowest survival rates were observed in plantlets without mycorrhizae during the drought stress period (Figure 2).

### 2.3. Mycorrhizal Characterization

**Figure 3:**
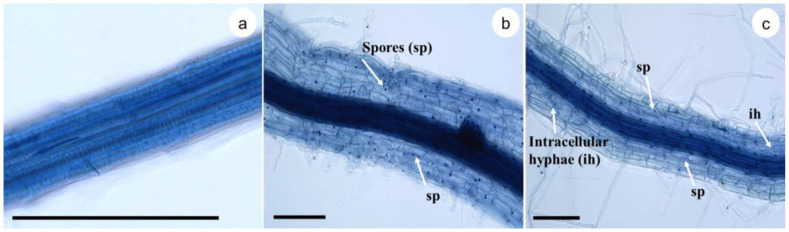
Colonization of sugarcane plantlets (*Saccharum* spp. cv Mex 69-290) with *Glomus intraradices* 60 d after spore inoculation. Fungal structures, such as spores (sp) and intracellular hyphae (ih), were observed by bright-field microscopy. (**a**) Control treatment, (**b**) 100 spores per plantlet, and (**c**) 200 spores per plantlet. Black Bar = 150 μm.

The microscopic analysis demonstrated the symbiotic relationship between plant and fungus with the presence of spores and hyphae (Figure 3). Spores were more frequently observed in the periphery of the root, between the epidermis and the mucilage that covers it. On the other hand, hyphae were observed internalized between the central cylinder and the parenchyma. We also observed arbuscular structures inside the cells, forming extensions of the hyphae outside the cell (Figure 3b,c).

### 2.4. Chlorophyll and Carotenoid Content

**Figure 4:**
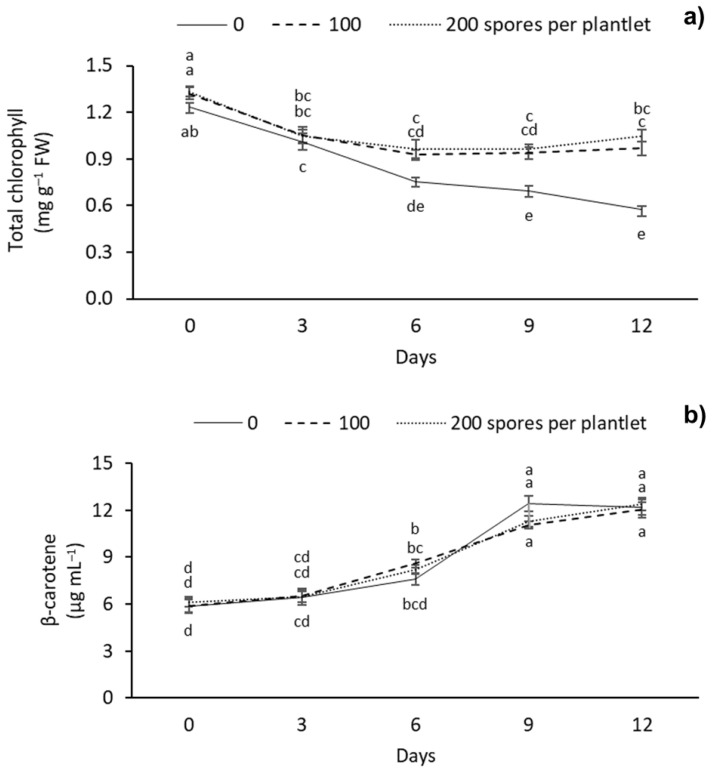
Effect of different doses (0, 100, and 200 spores per plantlet) of arbuscular mycorrhizal fungi (*Glomus intraradices*) on sugarcane plantlets (*Saccharum* spp. cv Mex 69-290) evaluated under drought stress during acclimatization stage. (**a**) Total chlorophyll and (**b**) β-carotene. Results are shown as mean ± standard error. Means with different letters (a–e) are significantly different (Tukey, *p* < 0.05).

Significantly different chlorophyll and carotenoid contents were observed between the different AMF doses evaluated (Figure 4). The highest chlorophyll content was observed at day 0 with 100 and 200 spores per plantlet with 1.32 and 1.33 mg g^–1^ FW, respectively. Meanwhile, the lowest levels of chlorophyll were observed in the treatment without mycorrhizae at 9 and 12 d of drought stress, with 0.69 and 0.57 mg g^–1^ FW, respectively (Figure 4a). As for the carotenoid content, a gradual increase was observed with drought stress days, regardless of the AMF dose applied. The highest carotenoid content was observed after 9 and 12 days under stress, with 12.43 and 12.38 mg g^–1^ FW, respectively. The lowest carotenoid content was observed at 0–6 days, independently of the mycorrhizae dose applied, with 5.85, 6.45 and 7.58 mg g^–1^ FW, respectively (Figure 4b).

### 2.5. Protein, Proline, and Glycine-Betaine Content

**Figure 5:**
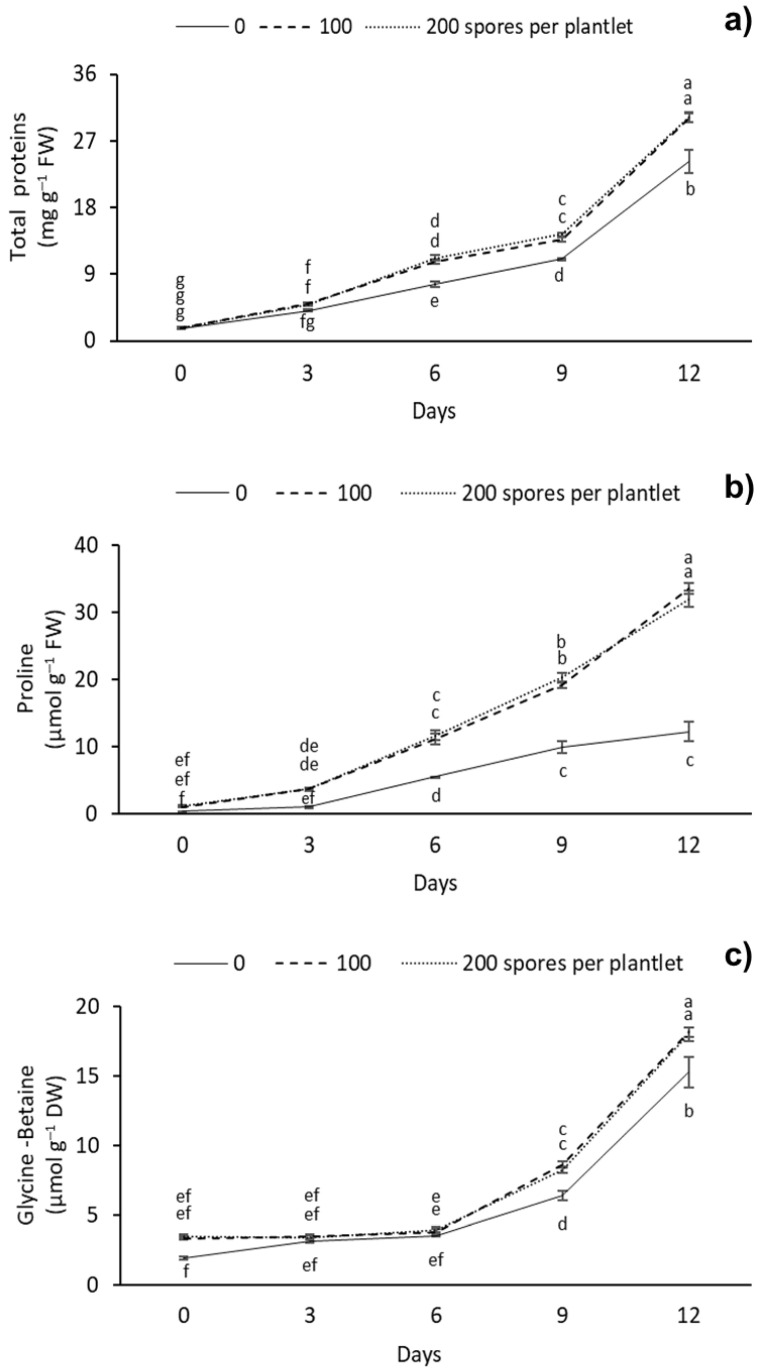
Effect of different doses (0, 100, and 200 spores per plantlet) of arbuscular mycorrhizal fungi (*Glomus intraradices*) on sugarcane plantlets (*Saccharum* spp. cv Mex 69-290) evaluated under drought stress during acclimatization stage. (**a**) Total protein, (**b**) proline and (**c**) glycine-betaine. Results are shown as mean ± standard error. Means with different letters (a–f) are significantly different (Tukey, *p* < 0.05).

TP, Pr, and GB contents significantly differed between the different AMF doses (Figure 5). Overall, a gradual increase in TP was observed as the drought stress days increased. The highest TP content was observed at 12 days with 100 and 200 spores per plantlets, with 30.14 and 30.21 mg g^–1^ FW, respectively. The lowest TP content was observed at day 0 in all of the doses evaluated, with 1.68 mg g^–1^ FW (Figure 5a). As for Pr, the highest levels of this amino acid were observed at 12 d with 100 and 200 spores per plantlet, with 33.56 and 32.05 mg g^–1^ FW, respectively. The lowest content was observed in the treatment without mycorrhizae at 0 days of drought stress, with 0.34 mg g^–1^ FW (Figure 5b). For GB, the highest content was observed at 12 d with the 100 and 200 spores per plantlet treatments, with 18.13 and 17.99 mg g^–1^ FW, respectively. The lowest GB content was observed at 0 d in the treatment without mycorrhizae, with 1.88 mg g^–1^ FW (Figure 5c).

### 2.6. Phenolic Content and Antioxidant Capacity

**Figure 6:**
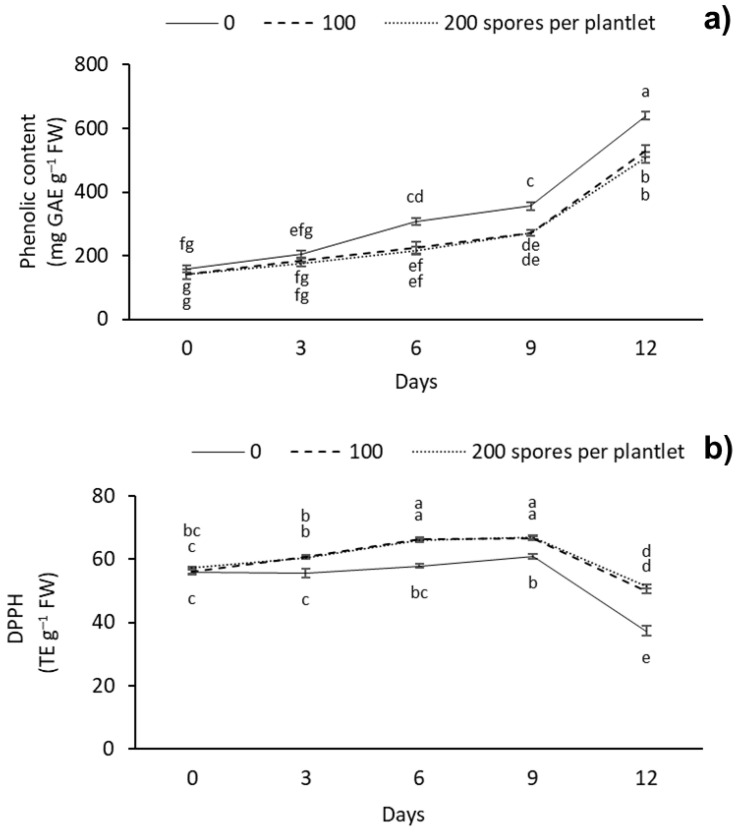
Effect of different doses (0, 100, and 200 spores per plantlet) of arbuscular mycorrhizal fungi (*Glomus intraradices*) on sugarcane plantlets (*Saccharum* spp. cv Mex 69-290) evaluated under drought stress during acclimatization stage. (**a**) Phenolic content expressed in GAE (milligrams of gallic acid equivalents per g of fresh weight) and (**b**) antioxidant capacity expressed in DPPH (2,2-Diphenyl–1-picrylhydrazyl), trolox equivalents (TE) per g of fresh weight. Results are shown as mean ± standard error. Means with different letters (a–g) are significantly different (Tukey, *p* < 0.05).

The phenolic content and antioxidant capacity between the different AMF doses and drought stress days were significantly different (Figure 6). At 12 d, the treatment without mycorrhizae showed the highest phenolic content, with 639.22 mg GAE g^–1^ FW, followed by the plantlets inoculated with 100 and 200 spores per plantlet, with 529.87 and 508.19 mg GAE g^–1^ FW, respectively. The lowest phenolic content was observed at day 0 with 100 and 200 spores per plantlet, with 140.21 and 140.75 mg GAE g^–1^ FW, respectively (Figure 6a). As for the antioxidant capacity, we observed that AMF treatment increased the antioxidant capacity of the plantlets during the different drought stress days evaluated. The highest content of DPPH was observed with the 100 and 200 spores per plantlet doses, with 66.32 and 66.12 TE g^–1^ FW at 6 days, and with 66.49 and 66.99 TE g^–1^ FW at 9 days of stress, respectively; the lowest antioxidant capacity was observed at 12 d in the treatment without mycorrhizae, with 37.37 TE g^–1^ FW (Figure 6b).

## 3. Discussion

### 3.1. Substrate Matric Potential Measurements

This study evaluated the effect of different AMF doses and drought stress days on the substrate matric potential. The substrate matric potential increased with the degree of drought stress. The effects of AMF on the matric potential have already been described in tomato (*Solanum lycopersicum* L.) [40], rice (*Oryza sativa* L.) [41], barrel clover (*Medicago truncatula*) [42,43], and wheat (*Triticum aestivum*) [44] crops. Bitterlich et al. [40] reported that S. lycopersicum plants inoculated with 72,500 spores of *Rhizoglomus irregulare* per plant had a water retention tolerance between –3 and –6 kPa. Chareesri et al. [41] observed that *O. sativa* plants inoculated with *Funneliformis mosseae*, *F. geosporus*, *Claroideoglomus claroideum*, *Glomus microaggregatum*, and *Rhizophagus irregularis* at a dose of 30 spores per plant had a lower yield when the substrate matric potential decreased to –40 and –80 kPa. Püschel et al. [43] studied *M. truncatula* plants inoculated with *Rhizophagus irregularis* at 780 spores per plant doses and reported that plant growth and nutrient acquisition decreased when the matric potential was lower than –100 kPa. Water deficit has negative effects on plant development. However, plants have biochemical and physiological strategies to maintain cellular homeostasis, such as improving their antioxidant system, chaperone functions to proteome maintenance, and osmotic adjustment to maintain cellular water content.

### 3.2. Survival Percentage and Mycorrhizal Characterization

Our results demonstrate that mycorrhizae affect the survival rate of plantlets subjected to drought stress. Overall, the survival rate decreased under stress conditions. However, plantlets treated with AMF had higher survival rates than controls on the different drought days evaluated. The positive effects of AMF on survival have been previously demonstrated in pear (*Pyrus communis*) [45], turmeric (*Curcuma longa* L.) [17], apple (*Malus prunifolia* (Willd.) Borkh.) [34], and common marigold (*Calendula officinalis* L.) [35]. Lotfi et al. [45] reported a survival rate of 95% in in vitro *P. communis* plantlets inoculated with 50 spores of *Rhizophagus irregularis* per plant, which was higher than the 70% survival rate observed in the control group. However, the variation in the survival rate of plants under drought stress and inoculated with AMF has already been reported on species such as *Cenostigma microphyllum* Mart. Ex G. Don [46] and the common myrtle (*Myrtus communis* L.) [47].

The survival rate of inoculated plants was higher than the control (100% vs. 30% survival). According to [48], mycorrhizae can store nutrients in their tissues while consuming photosynthetic products from plants, which could lead to nutrient competition. In this study, the lower survival rates obtained with higher AMF doses and drought stress degree could be due to the fungal hyphae, which reach more distant areas to obtain water and nutrients. Therefore, hyphae length increases soil volume compared to control plants. On the other hand, symbiosis with AMF improves the plant water state due to increased water absorption and improved root architecture derived from fungal colonization. The characterization of the mycorrhiza-sugarcane interaction demonstrated a symbiotic association between the two species, representing an advantage to the symbiotic interaction.

The mycorrhizae in this study formed ring-type arbuscules and scarce root mycelium. According to [49], the arbuscules are digested by host cells and then transformed into porous structures. We also observed small spherical sac-shaped spores in different areas, probably due to the apical expansion of the intraradical mycelium. Therefore, AMF can colonize the roots of plants and form intraradical hyphae, denser and finer than the root. Thus, hyphae help their host plants absorb more water and nutrients than non-mycorrhizal plants.

### 3.3. Total Chlorophyll and Carotenoid Content

In this study, the different doses of AMF affected the total chlorophyll and carotenoid content of sugarcane plantlets subjected to drought stress. During the stress period, plantlets treated with AMF had a higher chlorophyll content (75%) than the control. Meanwhile, the carotenoid content increased in all treatments during the drought stress period. The variation of chlorophyll and carotenoid content in plants under drought stress and inoculated with AMF has been previously reported in maize (*Zea mays*) [50], and thyme (*Thymus daenensis* Celak and *Thymus vulgaris* L.) [51]. Begum et al. [50] observed that the total chlorophyll content of *Z. mays* increased after inoculating with *Glomus versiform* (850 spores per plant) and a 4-week severe drought stress period. Arpanahi et al. [51] reported a decrease in the total chlorophyll content of *T. daenensis* and *T. vulgaris* after inoculation with *Rhizophagus intraradices* and *Funneliformis mosseaea* (3000 spores per plant) and a 3-week severe drought stress period. In *C. melo*, Meddich et al. [33] observed that the total chlorophyll content increased using a consortium of *Glomus* sp. at a dose of 44 spores per plant with partial root dehydration of eight weeks. The variation in chlorophyll content is an important factor that indirectly determines the photosynthetic capacity of plants. The plants growing under drought stress maintain a limited photosynthetic rate due to chlorophyll degradation and biosynthesis decrease. Therefore, low photosynthetic rates can interrupt carbon stabilization and, eventually, hinder plant development [52,53]. However, the increase in chlorophyll content after inoculation with AMF could be a drought tolerance mechanism that maintains photosynthetic metabolism [26,54].

As for the increase in carotenoid content, similar results have been reported by Begum et al. [50] in *Z. mays*. These authors observed an increase in carotenoid content after inoculation with *Glomus versiform* at a dose of 850 spores per plant and a 4-week drought stress period. In *C. melo*, Meddich et al. [33] reported an increase in carotenoid content with *Glomus* sp. at a dose of 44 spores per plant with partial root dehydration for eight weeks. Carotenoids are non-enzymatic antioxidants that target the excessive accumulation of ROS. Therefore, their increase could decrease photodegradation and photoinhibition and act as a drought tolerance mechanism [54]. Additionally, carotenoids are light scavengers for photosynthesis. They absorb light energy and transfer it to the chlorophylls at an absorption range in the 450–550 nm spectrum [55]. In this study, the increase in carotenoid content could contribute to maintaining photosynthetic activity during drought stress periods, in addition to an increase in antioxidant capacity.

### 3.4. Protein, Proline, and Glycine-Betaine Content

In this study, the different doses of AMF had an effect on the TP, Pr, and GB content of sugarcane plantlets subjected to drought stress. The accumulation of TP, Pr, and GB in plants under drought stress and inoculated with AMF has been previously reported in white clover (*Trifolium repens* L.) [56], thyme (*Thymus daenensis* Celak and *Thymus vulgaris* L.) [51], and woody plant (*Cinnamomum migao*) [36]. In this study, the TP content increased 80% as plantlets were exposed to stress with or without AMF. However, AMF-treated plants showed an increase in TP content during the different days of stress. Similarly, in *C. melo*, Meddich et al. [33] observed a significant increase in protein content after treatment with a *Glomus* sp. consortium (44 spores per plant) in plants under drought stress with 50% root dehydration for eight weeks. On the other hand, Liao et al. [57] observed that mycorrhizae (*Glomus lamellosum* and *Glomus etunicatum*) at different doses (0, 60, 120, 180, and 240 spores per plant) and a 2-week period of drought stress had no significant effect on the protein content of *C. migao*. The increase in protein content could be a drought stress tolerance response and represent the synthesis of hydrophilins, aquaporins (AQP), dehydrins, antioxidant enzymes, and chaperonins as tolerance mechanisms against drought stress [58]. AMF symbiosis specifically induces the expression of genes and protein transporters associated with a drought stress tolerance response; these include AQPs, inorganic phosphorus transporters, and ammonium, nitrate, sulfur, zinc, and carbon transporters [7,59,60].

Similar to TP, an increase in Pr and GB content was observed in sugarcane plantlets after prolonged drought stress. The highest Pr and GB contents (33% and 75%) were observed in plants treated with AMF. This effect confirms that Pr and GB are biochemical indicators contributing to sugarcane drought stress tolerance mechanisms. Similar to our study, Abd-Elghany et al. [37] observed that the Pr and GB content of *O. basilicum* increased after treatment with *Glomus versiform* (850 spores per plant) and a 4-week irrigation regimen at a 40% field capacity. To date, the accumulation of GB in sugarcane plantlets under drought stress and inoculated with AMF has not been reported. However, GB accumulation has been observed in other species subjected to drought stress. Begum et al. [50] observed that the GB content of *Z. mays* increased after inoculating *Glomus versiform* (850 spores per plant) and a 4-week severe drought stress period.

Drought stress tolerance is associated with Pr and GB accumulation. Pr is used as a biomarker of drought stress. This osmolyte, which acts as a chaperon protein, protects the cell through osmoregulation, maintains turgidity, and maintains the physiological and enzymatic activity of the plant. The AMF symbiosis can alter the gene expression of Δ1-pyrroline-5-carboxylate synthetase (P5CS) and protect the host plant from drought [6]. Meanwhile, GB acts as a compatible osmolyte and promotes antioxidant activity [5]. Furthermore, GB can protect the enzyme activity of Rubisco and photosystem II during photosynthesis and maintain membrane stability and cellular osmotic adjustment [61].

The AMF symbiosis in plants under drought stress could have induced the synthesis and regulated osmolyte catabolism, which resulted in the significant increase and accumulation of Pr and GB. Pr and GB accumulation could be due to the accumulation of soluble nitrogen compounds and free polyamines (PAs) induced by the AMF in plants under drought conditions. The PAs can bind anionic macromolecules such as nucleic acids and proteins to regulate transcription and translation, as well as maintain membrane stability and modulate antioxidant systems [62].

### 3.5. Soluble Phenols and Antioxidant Capacity

These results show the effect of the different doses of AMF on the soluble phenolic content and antioxidant capacity of sugarcane plantlets. The accumulation of soluble phenolic compounds and antioxidant capacity in plants inoculated with mycorrhiza has been reported in trees (*Tamarix gallica*) [63], globe artichoke (*Cynara cardunculus* L. cv scolymus Fiori) [64], prickly pear cactus (*Opuntia ficus-indica*) [65], and lettuce (*Lactuca sativa* L.) [66]. Bencherif et al. [63], in *T. gallica*, found that when using a consortium (*Funnneliformis mosseae*, *Septoglomus constrictum*, *Gigaspora gigantea*, *Glomus* sp1 and *Glomus* sp2) at a dose of 165 spores per plant, total phenolic content increased significantly more in roots than in leaves. Lahbouki et al. [65] in *O. ficus-indica* observed that when using a mycorrhizal consortium distributed into 22 species at a dose of 344 spores per plant, total phenolic content increased significantly, and antioxidant capacity decreased.

In this study, the soluble phenolic content increased 80% as the plantlets were subjected to increased drought stress. However, the soluble phenols content was lower when plantlets were inoculated with AMF. In response to water stress, plants can display some biochemical mechanisms that are independent of symbiotic interaction, such as synthesis of osmolytes, antioxidant capacity, and higher production of phenolic and flavonoid compounds [9]. Phenolic and flavonoid play key roles in protecting plants from excessive ROS production [9]. In AMF-colonized plants, AMF probably mitigates negative effects of drought stress through different mechanisms such as antioxidant defense systems, water absorption by extraradical hyphae and plants aquaporins, and up-regulation of antioxidant enzymes [62]. The increase in soluble phenols after inoculation with AMF and under drought stress has been reported in trifoliate orange (*Poncirus trifoliata* L. Raf.) [67], basil (*Ocimum basilicum* L.) [68], tobacco (*Nicotiana tabacum* L.) [50], and soybean (*Glycine max* L. Merril) [69]. Cheng et al. [67] in *O. basilicum* observed that when using *Funneliformis mosseae* at a dose of 2,200 spores per plant and a severe 8-week drought stress period, total phenolic content increased significantly. Sheteiwy et al. [69] in *G. max* found that when using a consortium with *Acaulospora laevis*, *Septoglomus deserticola*, and *Rhizophagus irregularis* at a dose of 250 spores per plant and a severe 2-week period of drought stress, total phenolic content increased significantly. The increase in soluble phenolic content could be used as a biomarker of drought stress. Phenolic compounds, such as phenolic acid, coumarin, xanthones, and flavonoids, are easily oxidized, producing free radicals and cell death. On the other hand, phenolic compounds and antioxidants eliminate free radicals, such as ascorbate, glutathione, hydrogen peroxide, and nicotinamide adenine dinucleotide phosphate (NADPH) [70]. Phenolic compounds can eliminate the reactive oxygen intermediates while preventing the initiation of other oxidative processes [71]. In addition, phenolic compounds are characterized by the availability of phenolic hydrogens as scavengers of hydrogen-donating radicals and, consequently, an increase in antioxidant capacity for scavenging activity [65].

As for the antioxidant capacity, we observed an increase (80%) in DPPH until day 9 (–53 kPa), which was higher in all the AMF treatments. However, the antioxidant capacity decreased on day 12 of drought stress, with a water potential of –199 kPa. This study indicated that *G. intraradices* could increase the antioxidant activity to resist the oxidative stress induced by drought stress. This was beneficial for the vitality of the AMF-inoculated plants, which might be partly due to the higher proline level because proline also plays an important role in ROS detoxification. Nahuelcura et al. [72] observed a significant increase in antioxidant capacity after inoculating wheat (*Triticum aestivum* L.) plants with 6000 *Funneliformis mosseae* spores per plant while subjected to a severe six-month drought stress period. Mohammadi et al. [73] in buckwheat (*Fagopyrum esculentum* Moench) observed that when using a mixture (1:1:1) of mycorrhizal fungi of *Rhizophagus fasciculatus*, *Funneliformis mosseae*, and *Rhizophagus irregularis* at a dose of 525 spores per plant and a 2-month severe drought stress period, the total antioxidant capacity increased significantly. The accumulation of phenolic compounds and the antioxidant capacity are mechanisms against oxidative stress. In this study, the drought stress tolerance induced by mycorrhizae could be associated with the antioxidant capacity.

In this study, drought stress tolerance is associated with the accumulation of photosynthetic pigments, Pr, GB, phenolic compounds, and antioxidant capacity. Previous studies have demonstrated that the fungus-plant symbiosis specifically induces gene expression (metallothionein (*MT*), *Cu/Zn SOD*, and *AQP*), aquaporin (AQP) activation, and enzymatic and non-enzymatic antioxidants, which are related to drought stress tolerance [74]. In addition, AMF protect host plants against drought stress through different mechanisms, including direct water absorption of the mycorrhizal extraradical mycelium and soil structure improvement by the mycorrhizal extraradical mycelium [62]. Sugarcane plantlets exposed to drought stress showed a low survival rate compared to those inoculated with AMF. Overall, plantlets inoculated with AMF had improved tolerance to drought stress via different physiological and biochemical aspects.

## 4. Materials and Methods

### 4.1. Plant Material and Micropropagation

For in vitro establishment of sugarcane, 25 cm apices of the Mex 69-290 cultivar were collected at eight months. These were cut to a length of 15 cm and subjected to hydrothermotherapy in a thermostatic bath (Ecoshel, SC-15, McAllen, TX, USA) at 50 °C for 20 min. Apices were reduced to 2 cm and rinsed for five min in a 10% (v/v) commercial sodium hydrochloride solution (5% of a.i.) (Cloralex™, Industrias Alen S.A. de C.V., Nuevo León, Mexico) with three drops of Tween 20^®^ (Sigma-Aldrich^®^ Chemical Company, Saint Louis, MO, USA) per 100 mL of water. Finally, meristems were extracted and placed in test tubes containing 10 mL of MS medium [75], without growth regulators. The medium pH was adjusted to 5.8, and 2.5 g L^−1^ of Phytagel™ (Sigma-Aldrich^®^) was added as a gelling agent. The medium was sterilized in an autoclave for 15 min at 120 °C and 115 kPa. The explants were incubated at 24 ± 2 °C, under 40 ± 5 μmol m^−2^ s^−1^ irradiance and a 16 h photoperiod. After one week, the apices were transferred for multiplication into MS medium supplemented with 1 mg L^−1^ kinetin (KIN, Sigma-Aldrich^®^), 1 mg L^−1^ indoleacetic acid (IAA, Sigma-Aldrich^®^), and 2 mg L^−1^ 6-benzylaminopurine (BAP, Sigma-Aldrich^®^). After four subcultures (45 d each), shoots were rooted in semi-solid MS medium without growth regulators.

### 4.2. Mycorrhizal Fungi Inoculation and Culture Conditions

Plantlets with a 5 cm length were inoculated with *Glomus intraradices* (Biofertilizante INIFAP^®^, Chiapas, MX) under ex vitro greenhouse conditions. Inoculation was carried out in 32-cavity polypropylene trays containing a substrate made up of compost, peat, and agrolite (2:1:1 *v*/*v*). Then, different doses of *G. intraradices* (0, 100, and 200 spores per plantlet) were added to the substrate and were homogenized. The substrate was sterilized in the autoclave for 30 min at 120 °C and 115 kPa. The inoculated plantlets were kept under greenhouse conditions with 60% shade at 30 ± 2 °C, relative humidity of 80 ± 10%, and natural light at an irradiance of 80 ± 10 μmol m^−2^ s^−1^ for a month. In a second phase, the dome that covered the plantlets was removed, which exposed them to temperatures of 35 ± 2 °C, relative humidity of 50 ± 10%, and natural light at an irradiance of 150 ± 10 μmol m^−2^ s^−1^ for a month. During the entire experiment, plantlets were irrigated with osmosis water twice a week for two months. After 60 days of greenhouse acclimatization, plantlets were subjected to 15 days without irrigation to simulate drought stress. During this period, we evaluated the survival rate. We also determined antioxidant capacity, total chlorophyll, carotenoids, total protein, proline, betaine glycine, and soluble phenolic compounds. Experiments were run in triplicate, inoculating 32 plantlets per replicate and *G. intraradices* dose.

### 4.3. Substrate Matric Potential Measurements

The substrate matric potential (Ψ) was determined every three days for 15 days by applying the tensiometer principle using an irrometer (IRROMETER Model SR, Riverside, CA, USA).

### 4.4. Mycorrhizal Characterization

Microscopy. Root segments were obtained to visualize the effect of the different doses of AMF on sugarcane plantlets. The segments were fixed in 4% paraformaldehyde and incubated for 48 h at room temperature. Root segments were washed three times with distilled water and incubated in 10% KOH for 15 min at 120 °C. Then, an alkaline hydrogen peroxide solution was added, followed by a 20 min incubation at room temperature, after which 0.05% trypan blue (Sigma-Aldrich^®^) was added and incubated for 24 h at room temperature. Finally, the trypan blue was removed, and an acetoglycerol solution was added. The samples were observed under a compound microscope (BX50, Olympus, Tokyo, Japan) using 20X/0.50, UPlan-FL (α−0.17), and 40x/1.00 (U-Plan-Apochromat, Olympus, Tokyo, Japan) objectives. Image acquisition was performed with an Infinity3 high-sensitivity fluorescence camera (Lumenera, Montreal, Canada) synchronized through Image Pro Premier 9.1 software (Media Cybernetics, Rockville, MD, USA).

### 4.5. Total Chlorophyll and Carotenoid Content

Total chlorophyll was determined following the methodology proposed by Harborne [76]. For each sample, 1 g of fresh matter (leaves) was macerated with 80% acetone and allowed to stand at –4 °C for 24 h in 80% acetone to a final volume of 10 mL. Then, the mixture was filtered through a No. 41 filter paper and adjusted to a 25 mL final volume with 80% acetone. Chlorophyll a and b content was determined in 2 mL aliquots by measuring absorbance at 663 and 645 nm, respectively. Absorbance measurements were carried out using a spectrophotometer (Thermo Fisher Scientific Genesys 10S, Madison, WI, USA).

Carotenoid content (β-carotene) was determined following the method proposed by Biehler et al. [77] and quantified using the following formula:

C= (A450 × M × 1000)/(ε × δ)

where: C = Carotenoid content; A = Absorption at 450 nm; M = β-carotene molecular mass (537 g^−1^ mol); ε = molar extinction coefficient of ß-carotene in acetone (140,663 L mol^–1^ cm^–1^); δ = optical path (cm).

### 4.6. Total Protein, Proline, and Glycine Betaine Content

Total protein (TP). TP was estimated following the method proposed by Bradford [78]. A sample of 20 mg of fresh plant material was weighed and macerated in a mortar with 25 mL of cold acetone. After adding 2.5 mL of 0.1 M tris-HCl buffer at pH 7.1, samples were placed on ice. Then, the solution was centrifuged at 3100× *g* for 20 min at 4 °C. Finally, after adding 5 mL of Bradford solution, absorbance at 595 nm was determined using a spectrophotometer (Thermo Fisher Scientific Genesys 10S, Madison, WI, USA). Quantification was done using a calibration curve with bovine albumin (Sigma-Aldrich^®^).

Proline (Pr) determination. Pr content was estimated according to the slightly modified colorimetric method by Bates et al. [79]. Samples containing 250 mg of fresh leaf tissue were macerated in a mortar and homogenized with 5 mL of 3% sulfosalicylic acid. Then, a 1-mL aliquot was taken, to which 1 mL of glacial acetic acid and 1 mL of ninhydrin (2.5% *w*/*v*) were added. This mixture was incubated in a thermoregulated bath for 1 h at 100 °C. Then, 2 mL of toluene was added. The upper phase (toluene + colored complex) was removed with a pipette. The absorbance of the resulting chromophore was read at 520 nm in the spectrophotometer (Thermo Fisher Scientific Genesys 10S, Madison, WI, USA). Absorbance values were interpolated in the calibration curve made with L-proline standard (Sigma-Aldrich^®^).

Glycine betaine (GB) determination. GB content was determined by following the colorimetric method proposed by Grieve and Grattan [80]. Samples of 250 mg of dry macerated plant tissue were suspended in 10 mL of deionized water. An aliquot of 0.5 mL diluted at a 1:1 ratio with 2 N H_2_SO_4_ was mixed with 0.1 mL of KI-I_2_ (35.7% *w*/*v*). Then, samples were stored at 0−4 °C for 16 h, centrifuged at 3,100 g for 15 min at 0 °C, and placed on ice for 1 h. Finally, the supernatant was collected, and 4.5 mL of 1,2-Dichloroethane was added; this solution was incubated at room temperature for 2 h. GB content was determined by measuring the absorbance of 3 mL aliquots at 365 nm (Thermo Fisher Scientific Genesys 10S, Madison, WI, USA). Measurements were interpolated in the calibration curve with glycine-betaine standard (Sigma-Aldrich^®^).

### 4.7. Soluble Phenolic Compounds and Antioxidant Capacity

Soluble phenolics. Phenolic content was determined according to Payet et al. [81]. First, 250 mg of fresh plant tissue was macerated in a mortar; extraction was performed with methanol: water (80:20). Then, the solution was centrifuged at 3100× *g* for 10 min at 10 °C. Subsequently, 150 µL of the supernatant was collected by adding 750 µL of 10% Folin-Ciocalteual reagent (E. Merck, Darmstadt, Deutschland); it was homogenized in a vortex (Corning^®^ LSE™), 600 µL of 20% Calcium Carbonate (Sigma-Aldrich^®^) was added and incubated for 2 h at 26 °C. Finally, the absorbance was measured at 765 nm using distilled water as a blank. Phenolic content was calculated from a gallic acid (Sigma-Aldrich^®^) calibration curve (0–10,000 µg mL^−1^) and expressed as milligrams of gallic acid equivalents (GAE) per g of fresh weight (g FW) of sugarcane plantlets.

Antioxidant capacity. The antioxidant capacity was expressed in DPPH (2,2-Diphenyl–1-picrylhydrazyl). The DPPH was performed by the methodology proposed by Huang et al. [82]. Briefly, 100 µL of the methanolic extract obtained in the soluble phenolics assay was added to 2900 µL of DPPH (0.0048% *w*/*v*). The mixture was incubated at 30 °C for 1 h; absorbance was measured at 515 nm. A calibration curve with Trolox (Sigma-Aldrich^®^) was used at different concentrations. The antioxidant capacity is expressed as Trolox equivalents (TE) per g of fresh weight (g FW) of sugarcane plantlets.

### 4.8. Experimental Design and Statistical Analysis

All experiments were performed following a completely randomized design and were run in triplicate. An analysis of variance was performed, followed by Tukey’s mean comparison (*p* < 0.05) using IBM SPSS^®^ statistical software (version 22 for Windows). The percentage data were transformed with the formula Y = arcsine (√ (×/100)), where × is the value of the percentage.

## 5. Conclusions

Together, our results demonstrate that mycorrhizae have an effect on the survival rate of plantlets subjected to drought stress. Survival tends to decrease as drought stress increases. However, plantlets inoculated with AMF maintained a higher survival rate. TP, Pr, GB, phenolic content, and antioxidant capacity help assess the stress level of plants and can be used for the early selection of drought-tolerant sugarcane cultivars. These tolerant mycorrhizal plants could be further treated with this low-cost and eco-friendly bioproduct for alleviating drought stress to improve sugarcane productivity. No significant changes in the different biochemical variables were noticed between treatments with 100 and 200 spores per plantlet. Therefore, the early inoculation of 100 spores of *G. intraradices* per sugarcane plantlet during the acclimatization stage of micropropagation could represent a preconditioning advantage before transplantation into the field and for establishing basic seedbeds.

## Data Availability

Not applicable.
